# Metabolic regulation by the intestinal metformin-AMPK axis

**DOI:** 10.1038/s41467-022-30477-3

**Published:** 2022-05-23

**Authors:** Song-Yang Zhang, Tony K. T. Lam

**Affiliations:** 1 Toronto General Hospital Research Institute, UHN, Toronto, ON Canada; 2 Department of Physiology, University of Toronto, Toronto, ON Canada; 3 Department of Medicine, University of Toronto, Toronto, ON Canada; 4 Banting and Best Diabetes Centre, University of Toronto, Toronto, ON Canada

**Keywords:** Endocrine system and metabolic diseases, Metabolism

## Abstract

AMP-activated protein kinase (AMPK) mediates the glucose-lowering effect of the antidiabetic agent metformin, but the sites of action remain unclear. In the March issue of Nature Communications, Zhang and colleagues reported that intestinal epithelium-specific AMPKα1 knockout mice fail to respond to metformin and exhibit disruption in metabolic homeostasis secondary to changes in the gut microbiome. This highlights a therapeutic potential of targeting intestinal AMPK for diabetes.

Metformin is a first-line glucose-lowering agent for people with obesity-associated type 2 diabetes, but the underlying mechanisms remains elusive. The liver has been described to mediate metformin action through AMPK-dependent and independent pathways^1,2^, but emerging studies highlight the gut as a target of metformin as well^3^. For example, oral delivery of metformin in rats and humans that targets the gut lowers plasma glucose levels independently of changes in plasma metformin levels^4,5^, while direct delivery of metformin into the upper small intestine of rats and humans also lower plasma glucose levels in diabetic conditions^6,7^. Thus, studies are urgently needed to identify molecular targets in the gut that are sufficient and necessary for metformin to regulate metabolic homeostasis.

Glucose homeostasis and energy balance in rodents and humans are regulated by nutrient sensing mechanisms in the small intestine^8^. AMPK is expressed in the small intestinal mucosa^6^ and, together with hepatic AMPK, is a known target of metformin. These findings raise the question of whether intestinal AMPK activation is sufficient and necessary for metformin to not only regulate glucose homeostasis, but also energy intake and expenditure as well as body weight in rodents and humans. In this regard, metformin has been documented to activate AMPK in the small intestine and to acutely and rapidly lower hepatic glucose production and plasma glucose levels in high-fat fed, obese and/or diabetic male rats^6^. But, whether small intestinal AMPK can exert metabolic control beyond glucose homeostasis such as energy intake and expenditure, and whether the gut metformin-AMPK axis has long-term effects on glucose levels and body weight in rodents and humans warrant investigation.

## Phenotypes of intestinal-specific AMPKα1 knockout mice

**Fig. 1:**
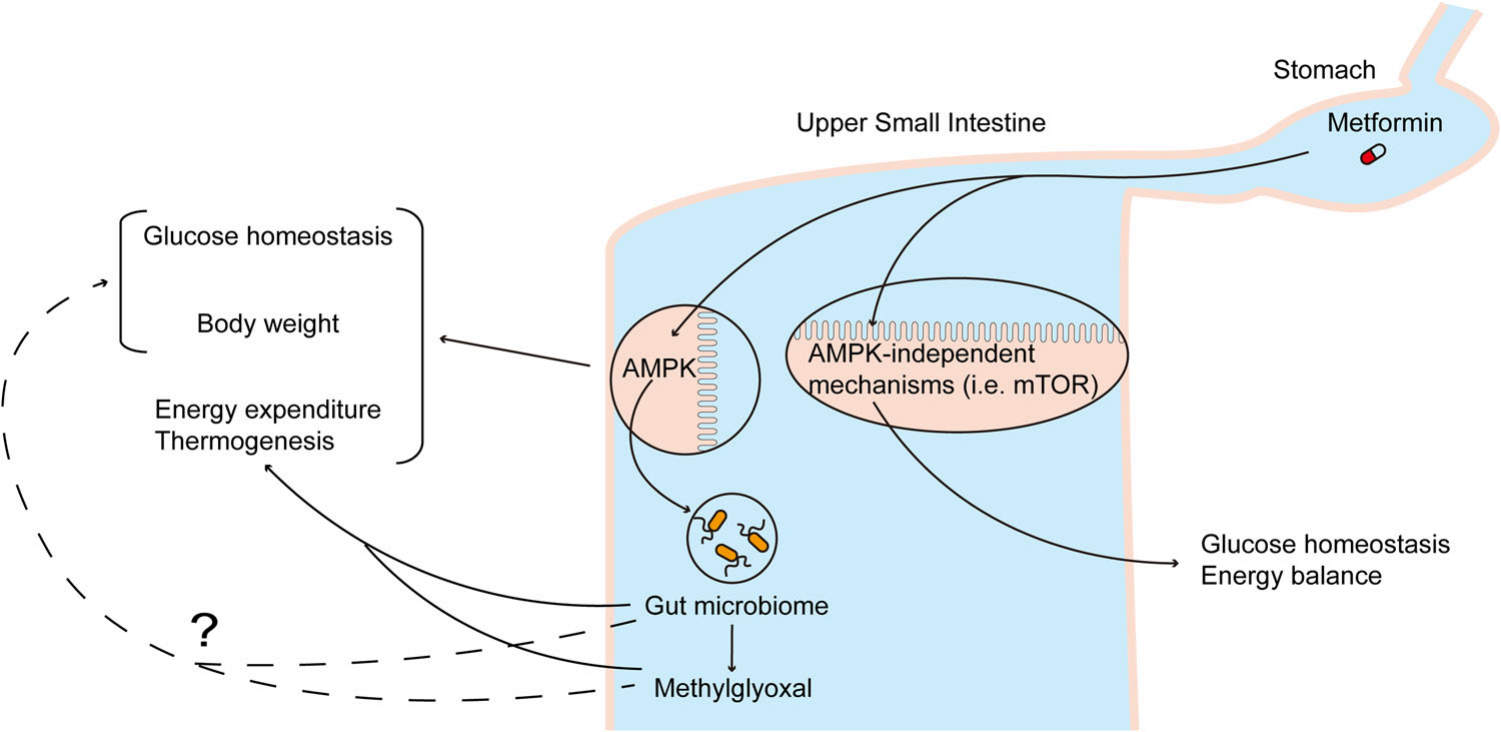
Metabolic regulation by metformin through intestinal AMPK-dependent and -independent pathways. With the use of high-fat fed intestinal AMPKα1 knockout mice, Zhang et al. demonstrated that intestinal AMPKα1 deficiency per se altered energy expenditure and brown fat thermogenesis through changes in the gut microbiome and potentially methylglyoxal, impaired glucose tolerance and induced weight gain. Intestinal AMPKα1 deficiency also impaired the ability of metformin to increase glucose tolerance and lower body weight. In parallel, metformin alters intestinal AMPK-independent pathways (i.e., mTOR) to regulate glucose homeostasis. We propose metformin activates small intestinal AMPK-dependent and –independent pathways to regulate glucose homeostasis and energy balance in diabetes and obesity.

In March, *Nature Communications* published the work of Zhang and colleagues^9^, who generated intestinal epithelium-specific AMPKα1 knockout mice and found that after 6 weeks of high fat feeding and in comparison to wild-type mice, these mice exhibit weight gain that is independent of hyperphagia, accompanied by impaired glucose tolerance, as assessed by a intraperitoneal glucose injection test. The disruption of glucose tolerance was found to be independent of weight gain but occurred in parallel to increased expression of hepatic genes that regulate gluconeogenesis^9^. Importantly, once daily (100 mg/kg) oral metformin administration for 8 weeks lowered weight and increased glucose tolerance independent of weight changes in 6 week high-fat fed wild-type but not in intestinal AMPKα1 knockout mice^9^. These studies highlight that metformin activates intestinal AMPK to improve glucose tolerance and lower body weight together with its lowering effect on hepatic glucose production^6,9^, and chronic inhibition of intestinal AMPK is sufficient to dysregulate glucose homeostasis and induce obesity (Fig. 1**)**. Although the clinical relevance remains to be directly tested, preliminary findings indicate that AMPK activity was reduced in the upper small intestine of people with obesity-associated type 2 diabetes as well^9^.

An equally important discovery was that intestinal AMPKα1 knockout mice on chow diet develop adipocyte hypertrophy with a downregulation of the brown fat thermogenic program (i.e., reduced UCP1 expression) and a subsequent reduction in energy expenditure^9^, that likely resulted in greater obesity upon high fat feeding. The effect of intestinal AMPKα1 knockout mice on the brown fat thermogenic program was reproduced by Zhang et al. in mice that received fecal microbiota transplant from intestinal AMPKα1 knockout mice, altogether demonstrating a novel intestinal AMPK-microbiome-brown fat axis that regulate energy expenditure (Fig. 1). Consistent with the fact that microbiota-derived metabolite methylglyoxal is higher in people with diabetes, methylglyoxal was higher in the serum, fecal and brown fat of high-fat fed intestinal-specific AMPKα1 knockout vs. wild-type mice^9^. In fact, methylglyoxal administration reduced brown fat UCP1 expression in vitro and in vivo^9^, but whether changes in methylglyoxal is responsible for the reduction in energy expenditure in intestinal-specific AMPKα1 knockout mice remain unclear (Fig. 1). In addition, the specific microbes families and/or species that were involved remain unknown, although the relative abundance of the *Lachnospiraceae* family was significantly lower^9^. In summary, this elegant set of studies^9^ significantly advances and expands the understanding of the metabolic regulation by intestinal AMPK (Fig. 1).

## Perspective and future directions

Although the current study illustrates metformin activates small intestinal AMPK to regulate glucose homeostasis, intestinal AMPK-independent pathways are also necessary for metformin action (Fig. 1). For example, metformin inhibits upper small intestinal mTOR to lower plasma glucose levels and hepatic glucose production independent of changes in gut AMPK^10^. Second, metformin induces changes in gut microbiome to inhibit small intestinal bile acid receptor FXR independent of gut AMPK to improve glucose homeostasis^11^. Third, metformin-induced changes in gut microbiome enhances upper small intestinal glucose sensing via sodium glucose cotransporter-1 to lower hepatic glucose production^12^ possibly via FXR inhibition, as direct small intestinal inhibition of FXR enhances gut glucose sensing to increase intravenous glucose tolerance^13^. If one considers the fact that gut glucose sensing is not required for upper small intestinal metformin-AMPK axis to lower glucose production^8^, it is reasonable to postulate that the upper small intestinal glucose-sodium glucose cotransporter-1 axis that facilitate metformin-mediated gut microbiota changes to lower hepatic glucose production is gut AMPK-independent as well. Collectively, we put forward a working hypothesis that metformin activates small intestinal AMPK-dependent and -independent pathways to regulate glucose and energy homeostasis (Fig. 1).

AMPKα catalytic subunit has two isoforms, α1 and α2, which are both activated by metformin^1^. The relative contribution of AMPKα1 and α2 to metformin action, however, remains unclear. Interestingly, although the re-expression of either AMPKα1 or α2 in the liver of HF-fed liver-specific double AMPKα1 and α2 knockout mice still impair metformin’s ability to lower glucose production as compared to wild-type mice, the loss of hepatic AMPKα1 vs. AMPKα2 results in higher glucose production^14^, suggesting that hepatic AMPKα1 may have a dominant role for metformin action. In parallel, a recent study^15^ reports intestinal AMPKα1 and 2 double knockout vs. wild-type mice on chow diet displayed comparable body weight, fat mass and glucose tolerance similar to the intestinal AMPKα1 knockout mice^9^. In contrast, intestinal AMPKα1α2 knockout mice did not show changes in energy expenditure, thermogenesis, body weight, fat mass and glucose tolerance after ten weeks on a high fat diet^15^. Intestinal AMPKα1α2 knockout mice were co-housed with the wild-type mice in the same cages during the experiments that may have led to the exchange and homogenization of gut microbiome, although the gut microbiome was not assessed among the groups in the study^15^. A homogenization of the gut microbiome of AMPKα1α2 knockout with wild-type mice may have eliminated any potential adipocyte changes since the reduction in energy expenditure and thermogenesis detected in intestinal AMPKα1 knockout mice were gut microbiome-dependent^9^. Nonetheless, whether AMPKα1 vs. α2 in the intestine has additive, redundant, or even opposing metabolic roles remain to be investigated.

Metformin was equally effective in increasing glucose tolerance in high fat fed intestinal AMPKα1α2 knockout and wild-type mice^15^. Specifically, a single oral dose of metformin was administered to the intestinal AMPKα1α2 knockout mice shortly before an oral glucose tolerance test (OGTT)^15^, while long-term daily oral metformin administration was given to the intestinal AMPKα1 knockout mice that undergone an intraperitoneal glucose tolerance test (IPGTT)^9^. Given that an acute constant infusion of metformin into the upper small intestine of high-fat fed rats not only activates AMPK but also inhibits mTOR in an AMPK-independent fashion to lower hepatic glucose production^6,10^, it is possible that metformin was able to increase glucose tolerance in intestinal AMPKα1α2 knockout mice^15^ due to a concurrent inhibition of intestinal mTOR. On the other hand, long-term metformin administration may have desensitized the effect on intestinal mTOR, leading to the inability of metformin to increase glucose tolerance in intestinal AMPKα1 knockout mice^9^. Further, the use of OGTT that triggers gut glucose sensing in AMPKα1α2 knockout mice^15^ may have allowed metformin to increase glucose tolerance, as metformin activates AMPK independent of glucose sensing in the upper small intestine to lower glucose production^6^, consistent with the fact that metformin failed to increase glucose tolerance (assessed by IPGTT and not OGTT) in AMPKα1 knockout mice independent of gut glucose sensing. Nonetheless, the above speculations remain to be tested.

The study by ref. ^9^ points to the following questions for future research: are the changes in the gut microbiome and/or thermogenesis and energy expenditure incurred by intestinal AMPK-deficiency directly responsible for the induction of weight gain and/or glucose tolerance during high-fat feeding? Does metformin activate an AMPK-Reg3γ (or α) axis to regulate glucose tolerance, hepatic glucose production and body weight? Are changes in microbiota-derived plasma methylglyoxal responsible for the metabolic benefits of metformin action? What is the relative contribution of hepatic vs. extrahepatic (i.e., brown fat) glucose metabolism in mediating the glucose-lowering effect of the small intestinal metformin-AMPK axis? To begin answering these questions would require the use of genetic and chemical tools to address the cause-and-effect in vivo relationship for the respective mechanisms.

In summary, ref. ^9^ elucidated intestinal AMPKα1 deficiency in high-fat fed mice altered energy expenditure and thermogenesis of brown fat via changes in gut microbiome, impaired glucose tolerance and increased body weight. Further, intestinal AMPKα1 deficiency reduced the ability of metformin to increase glucose tolerance and lower body weight in response to high-fat feeding. These studies highlight the metabolic role and therapeutic potential of activating intestinal AMPK for the treatment of diabetes and obesity.
